# A steadily increasing trend in the incidence of esophageal adenocarcinoma in Akita Prefecture, Japan, through 2024

**DOI:** 10.1007/s00535-026-02407-3

**Published:** 2026-04-09

**Authors:** Kenta Watanabe, Hiroyuki Shibata, Katsunori Iijima

**Affiliations:** 1 Department of Gastroenterology, Akita University Graduate School of Medicine, 1-1-1 Hondo, Akita, 010-8543 Japan; 2 Department of Clinical Oncology & Center for Cancer Registry and Information Services, Akita University Graduate School of Medicine, 1-1-1 Hondo, Akita, 010-8543 Japan

**Keywords:** Esophageal adenocarcinoma, Age-standardized incidence rate, Annual percent change, Population-based registry, Sex differences

## Abstract

**Background:**

A population-based study of esophageal adenocarcinoma (EAC) incidence in Akita Prefecture (2007–2014) revealed a surge beginning around 2010. Using the same registry, we extended the analysis through 2024 to examine recent EAC trends in the prefecture.

**Methods:**

We used the Akita Prefecture collaborative, hospital-based cancer registry, which captures > 90% of cancers in the region. Registered esophageal cancers were histologically classified as squamous cell carcinoma, EAC, and unspecified neoplasm. Temporal changes in the number of and proportion of EAC cases, as well as the age-standardized incidence rates (ASRs; per 100,000 person-years, directly standardized to the 1985 Japanese model population), were estimated for 2010–2024. The estimated annual percent change (EAPC) was also calculated.

**Results:**

The number of EAC cases increased from 38 (2010–2014) to 72 (2020–2024), and the proportion among all esophageal cancers rose from 2.3% to 4.7%. The ASR increased from 0.39 (2010–2014) to 0.45 (2015–2019) and 0.77 (2020–2024) per 100,000 person-years. From 2010 to 2024, ASR showed a significant upward trend (EAPC 8.37% [95% confidence interval, 4.32–12.57]; *p* < 0.0001), largely driven by men.

**Conclusion:**

Using the most recent data from Akita’s hospital-based registry, we demonstrate that both the number and incidence of EAC approximately doubled between 2010–2014 and 2020–2024, indicating that the upward trend that began around 2010 has persisted.

**Supplementary Information:**

The online version contains supplementary material available at 10.1007/s00535-026-02407-3.

## Introduction

Esophageal cancer (EC) is histologically categorized into two major subtypes, esophageal squamous cell carcinoma (ESCC) and esophageal adenocarcinoma (EAC), each characterized by distinct epidemiological and etiological features [1, 2]. Globally, ESCC had long been the predominant subtype; however, in Western countries, EAC began to rise in the 1960 s and 1970 s [3, 4], followed by a sharp increase during the 1980 s and a subsequent plateau in the 2000 s and 2010 s [5–9]. As a result, EAC eventually surpassed ESCC in incidence, highlighting the growing clinical importance of strategies specifically targeting EAC prevention and management [10, 11].

In Japan, ESCC still accounts for the vast majority (80–90%) of EC. Nevertheless, multiple studies, including our previous report, have demonstrated that the incidence of EAC has been increasing since around 2010 [12–15], approximately 50 years later than in Western countries [16]. Considering the historical trajectory observed in Western populations, understanding the subsequent trends in EAC incidence in Japan is of particular interest. However, all previous epidemiological reports from Japan have been limited to data up to 2014 [12–15], leaving more recent trends unclear.

Accurate assessment of longitudinal incidence patterns requires a stable underlying population and reliable, population-based cancer registration. Akita Prefecture, located in northern Honshu with a current population of approximately 900,000, is characterized by limited interregional migration owing to its economy being largely dependent on traditional industries such as agriculture, fishing, and forestry. This demographic stability makes Akita Prefecture an optimal setting for the evaluation of temporal cancer trends [12].

A hospital-based, collaborative cancer registration system was launched in 2007, covering more than 90% of all newly diagnosed cancers and providing timely population-based data for the region [12, 17]. Using this database, we previously reported a marked increase in EAC in Akita Prefecture beginning around 2010 [12]. In the present study, we extend our analysis by incorporating data updated through 2024 to clarify the subsequent trajectory of EAC incidence in this region.

## Methods

We utilized data from the collaborative, hospital-based cancer registry system in Akita Prefecture [12.17]. All regional cancer care hospitals in the prefecture participate in this system. The registry was launched in 2007 with five hospitals, including Akita University Hospital, and the number of participating institutions gradually increased to 11 in 2011; since 2016, 12 hospitals have consistently contributed data. Newly diagnosed cancer cases were systematically registered regardless of subsequent treatment, including endoscopic resection, surgery, chemoradiotherapy, or palliative care. This registration system provides data several years earlier than the official population-based cancer registry and ultimately captures more than 90% of all cancers occurring in Akita Prefecture [12, 17]. Therefore, this database serves as a reliable, timely, population-based data source. For the current analysis, we extracted all cases of EC registered in the system.

Registered EC cases were categorized according to ICD-O-3 morphology codes into the following groups: ESCC, EAC, and unspecified neoplasm. The classification criteria remained unchanged throughout the study period [12]. Demographic information (age and sex) and clinical stage at diagnosis were also obtained from the registry.

This study was reviewed and approved by the ethics committee of Akita University (No. 2796).

### Statistical analysis

We first summarized the annual number of ESCC and EAC cases and calculated the proportion of EAC among all ECs across the 18-year study period. Because EAC incidence was relatively low and subject to year-to-year fluctuation, the study period was divided into four intervals: the initial 3-year period (2007–2009), followed by three consecutive 5-year periods (2010–2014, 2015–2019, and 2020–2024). Comparative analyses were performed across these intervals, and a period-based formal trend was assessed using the Cochran–Armitage trend test applied to period-specific EAC incidence proportions.

The crude incidence rate (IR) and age-standardized incidence rate (ASR) for EAC were calculated for each study period. Incidence per 100,000 persons was estimated annually by sex and age, and age adjustment was performed using the 1985 Japanese model population with direct standardization, applying observed age-specific rates to the standard population [14].

Temporal trends in IRs were evaluated using estimated annual percent change (EAPC), with corresponding 95% confidence intervals (CIs) [14]. Continuous variables were presented as mean (standard deviation) and compared using Student’s *t test*. In a supplementary, exploratory analysis, we also fitted joinpoint regression models (log-linear; 0–1 joinpoints; weighted BIC for model selection) to annual ASRs (overall and by sex) [18]. Statistical analyses were performed using EZR (Saitama Medical Center, Jichi Medical University, Saitama, Japan), a graphical user interface for R (The R Foundation for Statistical Computing, Vienna, Austria) [19] and the NCI Joinpoint Regression Program. A two-sided *p-value* < 0.05 was considered statistically significant.

## Results

During the 18-year study period, a total of 5974 EC cases were registered. Of these, 5621 (94.1%) were diagnosed as ESCC and 159 (2.7%) as EAC, with the remaining 194 cases (3.2%) classified as unspecified. Among the ESCC cases, 4888 were male, and 733 were female; among the EAC cases, 138 were male, and 21 were female. Thus, the male-to-female ratios for ESCC and EAC were similarly high at 6.7 and 6.6, respectively. The mean age at diagnosis showed minimal sex-related differences in ESCC [men: 69.9 (9.2) years vs. women: 70.9 (9.5); NS], whereas a marked age difference was observed in EAC, with men diagnosed at a significantly younger age than women [67.7 (10.8) years vs. 79.7 (11.0) years, *p* < 0.01].

### Temporal trends in ESCC and EAC

**Fig. 1:**
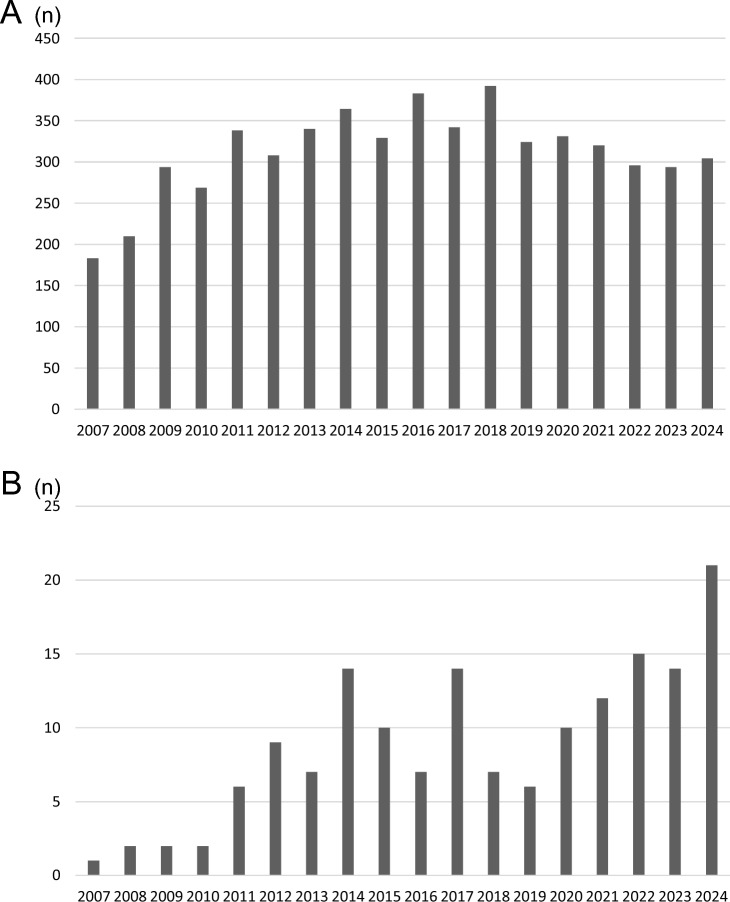
Annual number of esophageal squamous cell carcinoma **A** and esophageal adenocarcinoma **B** cases registered in Akita Prefecture, 2007–2024

Annual numbers of ESCC and EAC cases from 2007 to 2024 and their stage distributions are shown in Fig. 1 and Supplementary Fig. 1. From 2016 onward, when the number of participating hospitals stabilized at 12, ESCC cases demonstrated a slight downward trend, whereas EAC cases increased despite year-to-year variation. In the early years of the study, EAC was diagnosed only sporadically, but in the final years, the number consistently exceeded 10 cases annually (Fig. 1B).

**Fig. 2:**
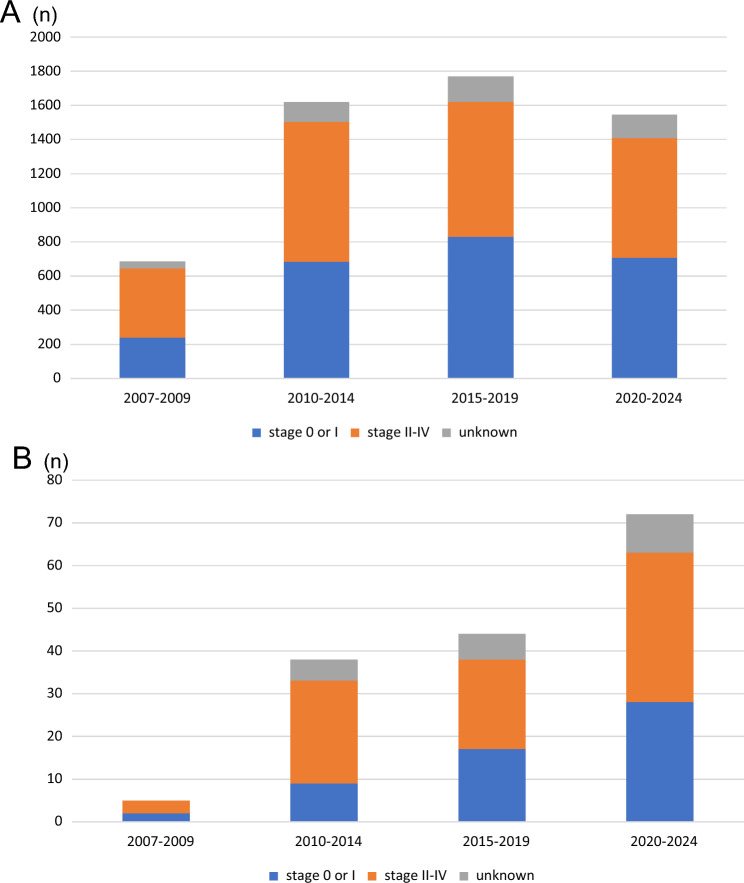
Changes in the number of esophageal squamous cell carcinoma (A) and esophageal adenocarcinoma (B) cases stratified by clinical stage (early- vs. advanced-stage) across the four study periods (2007–2009, 2010–2014, 2015–2019, and 2020–2024)

Figure 2 shows changes across four study periods (2007–2009, 2010–2014, 2015–2019, and 2020–2024) stratified by clinical stage. The number of ESCC cases increased from 2007–2009 to 2010–2014 and to 2015–2019, largely attributable to the expansion of participating hospitals. However, despite stable registry participation thereafter, ESCC cases decreased by 12.7% in 2020–2024 compared with 2015–2019 (Fig. 2A). By contrast, EAC cases showed a marked rise from 2007–2009 to 2010–2014, again partly reflecting increased hospital participation. Notably, our previous study also observed a significant increase in EAC cases in a subgroup analysis that confined the subjects to patients from the eight hospitals that participated in the registration continuously from 2009 to 2014 [12]. However, even after registry stabilization, EAC cases continued to increase in 2015–2019 and 2020–2024. Overall, the number of EAC cases in 2020–2024 was 1.9-fold higher than in 2010–2014 period. When stratified by stage, early-stage EAC increased 3.1-fold and advanced-stage EAC increased 1.5-fold during this 10-year interval, indicating a particularly prominent rise in early-stage detection (Fig. 2B).

### Temporal trends in the proportion of EAC

**Fig. 3:**
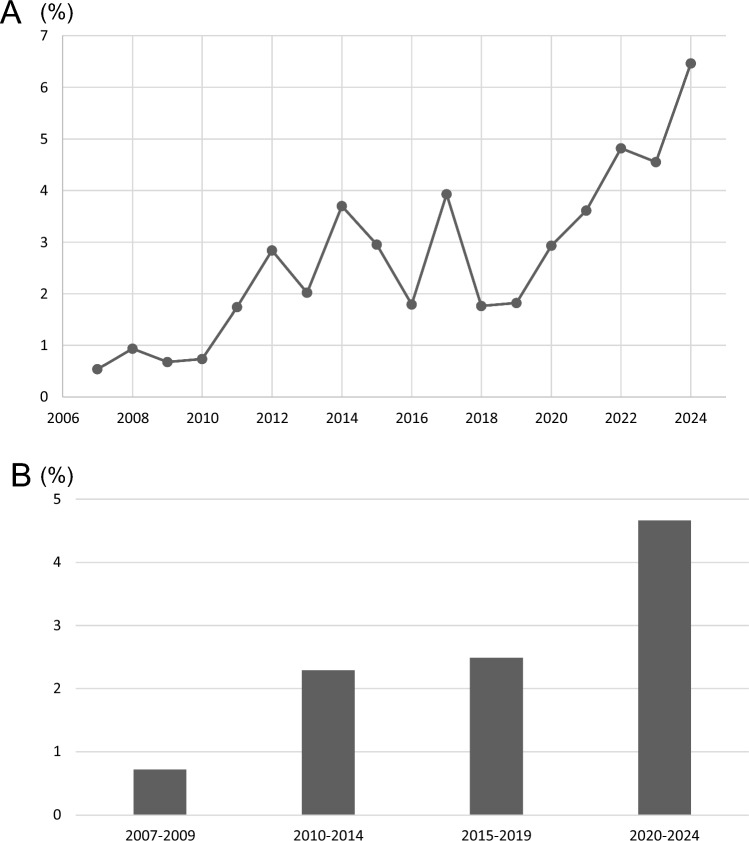
Trends in the proportion of esophageal adenocarcinoma among all esophageal cancer cases from 2007 to 2024. **A** Annual proportion. **B** Proportion across the four study periods (2007–2009, 2010–2014, 2015–2019, and 2020–2024)

Figure 3A illustrates the annual proportion of EAC among all EC between 2007 and 2024, rising from 0.5% to 6.4% despite fluctuations. Across the four study periods, the proportion increased steadily: 0.7% (2007–2009), 2.3% (2010–2014), 2.5% (2015–2019), and 4.7% (2020–2024). Thus, the proportion of EAC doubled over the last 10-year interval (2010–2014 vs. 2020–2024) (Fig. 3B). A formal period-based trend test using the Cochran–Armitage method confirmed a significant increase across 2010–2014, 2015–2019, and 2020–2024 in the total cohort and by sex (Supplementary Table 1).

### Temporal trends in incidence rates

Crude IRs and ASRs for the total population and by sex are presented in Table 1. Among the entire cohort, crude IR increased from 0.70 (95% CI: 0.49–0.96) per 100,000 person-years in 2010–2014 to 0.85 (0.62–1.15) in 2015–2019 and to 1.51 (1.18–1.90) in 2020–2024. Correspondingly, the ASR increased from 0.39 to 0.45 and to 0.77 per 100,000 person-years. The increase was largely driven by male subjects. In men, crude IR rose from 1.32 (0.92–1.85) to 1.57 (1.11–2.15) and to 2.70 (2.07–3.47) across the three periods, corresponding to ASRs of 0.75, 0.86, and 1.46, respectively. In women, the crude IR also showed an upward trend (from 0.14 to 0.22 and 0.44 per 100,000 person-years), but the number of cases was insufficient to yield a reliable ASR. In particular, age standardization for women was not performed when the 5-year count was < 10, and values should be interpreted as a reference only.

**Fig. 4:**
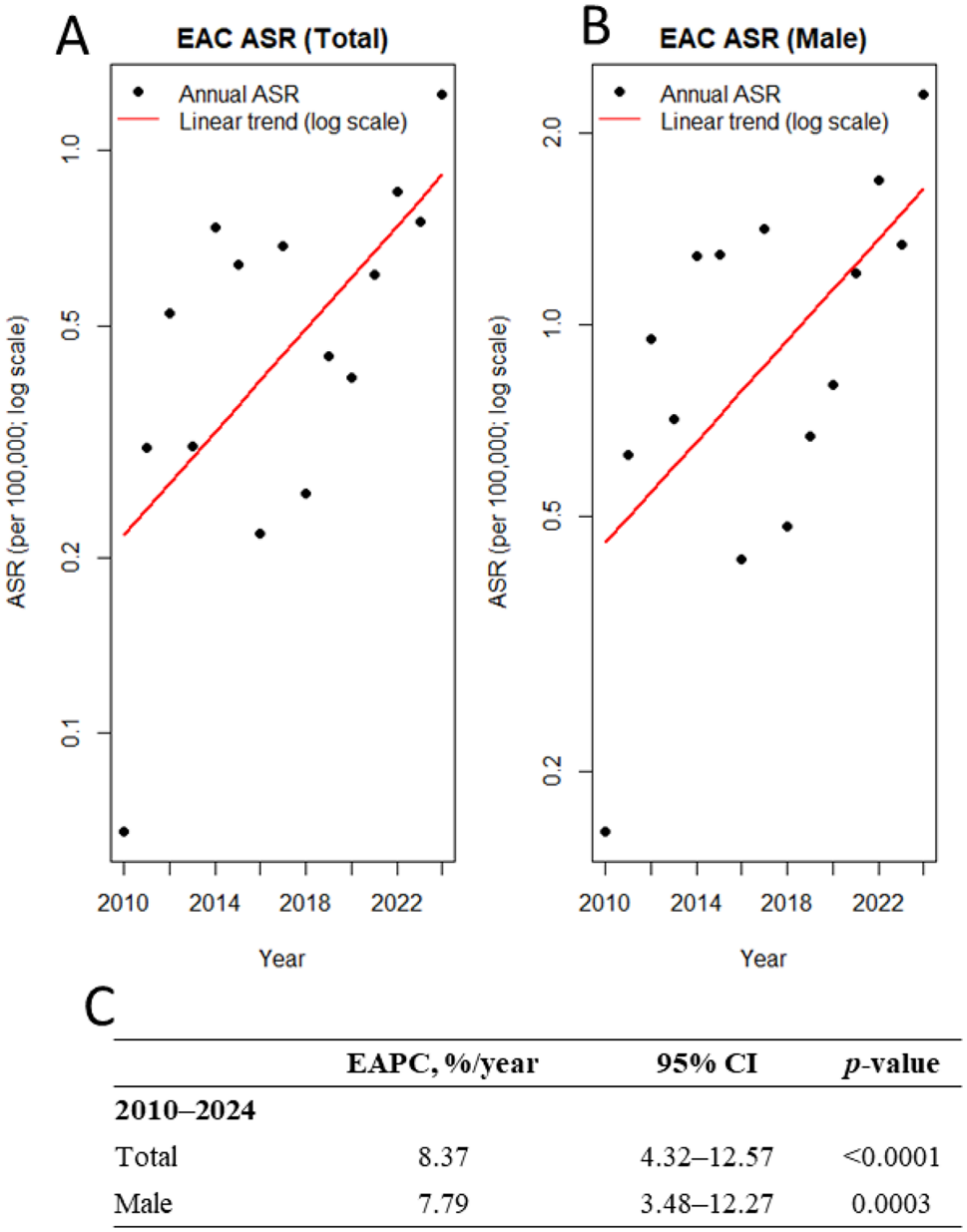
Age-standardized incidence rates of esophageal adenocarcinoma from 2010 to 2024. **A** Entire cohort. **B** Male subjects. **C** Estimated annual percent change (EAPC) with 95% confidence intervals. *ASR* age-standardized incidence rate, *CI* confidence interval, *EAPC* estimated annual percent change

Figure 4 shows annual ASR changes between 2010 and 2024. For the entire cohort, ASR increased significantly, with an EAPC of 8.37% (3.48–12.57; *p* < 0.0001). Similarly, the ASR among men exhibited a substantial increase during this period, with a statistically significant EAPC of 7.79% (3.48–12.27%) per year, *p* = 0.0003. In 2024, the final study year, the ASR among men reached 2.1 per 100,000 person-years. For visual context, annual female ASRs and an age-adjusted, Poisson regression-based EAPC are shown in Supplementary Fig. 2; given sparse counts and zero-ASR years, these results are provided as reference and should be interpreted with caution. In supplementary, exploratory joinpoint regression of annual ASRs (2010–2024), no joinpoints were selected for the overall or male series, supporting a monotonic increase; details are provided in Supplementary Fig. 3.

### Age distribution at diagnosis

**Fig. 5:**
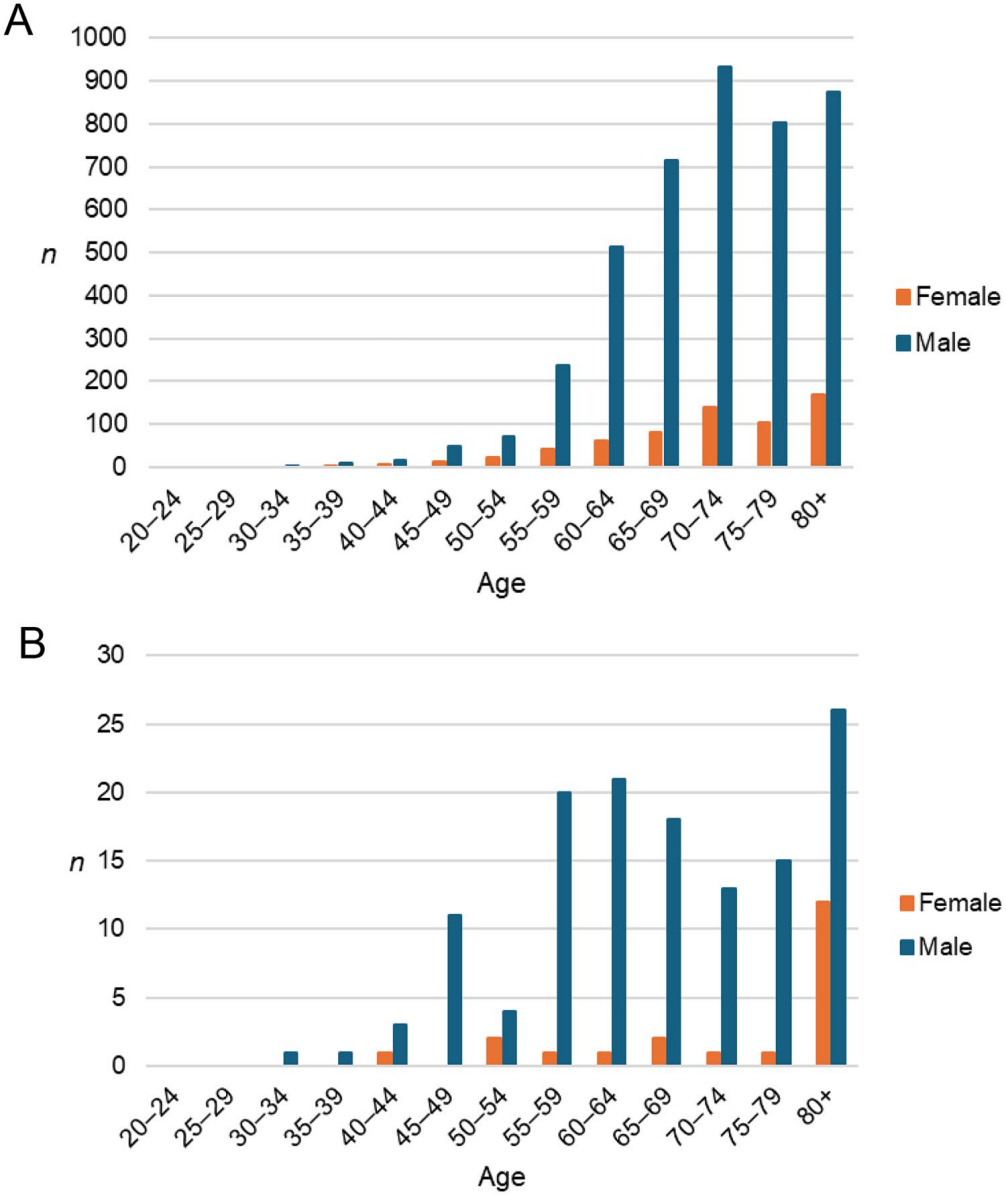
Number of esophageal squamous cell carcinoma **A** and esophageal adenocarcinoma **B** cases by age group and sex

Given the pronounced sex differences in mean age at EAC diagnosis, we examined age distributions stratified by sex (Fig. 5). For both ESCC and EAC, male predominance was consistent across all age groups. However, distinct age patterns emerged between sexes. In ESCC, age distributions were nearly identical for men and women (Fig. 5A). In EAC, cases in men were concentrated in the 50 s and 60 s, whereas in women, EAC was rare before age 70, with most cases diagnosed in the 80 s or later (Fig. 5B).

## Discussion

Using data from the hospital-based cancer registry system in Akita Prefecture, our previous report demonstrated that EAC was rare but began increasing around 2010 [12]. Subsequent studies from other Japanese regions confirmed a similar rise [13–15]. Previous reports, however, documented EAC trends only up to 2014 [12–15]. By extending the analysis to 2024, the present study shows that both the number and proportion of EAC cases doubled over the most recent 10-year interval (2010–2014 vs. 2020–2024). Furthermore, ASR increased significantly from 2010 to 2024, particularly among men. To our knowledge, this is the first study to document EAC trends in Japan through 2024.

In evaluating recent incidence trends, the potential impact of SARS-CoV-2 must be considered, as the pandemic may have reduced opportunities for medical evaluation, leading to missed cancer diagnosis [20–23]. Indeed, the same registry system used in this analysis showed a modest decline in early-stage gastrointestinal cancers in 2020 [24]. However, case numbers recovered in 2021 and returned fully to pre-pandemic levels by 2022 [25, 26]. Thus, the pandemic is unlikely to have substantially influenced the long-term trends in EC observed in the present study.

In Western countries, where the rise in EAC began roughly 50 years earlier than in Japan [16], a gradual increase during the initial 10–20 years was followed by a steep escalation beginning in the 1980 s [2–7]. For example, among men in England and Scotland, where increases occurred earliest, ASR rose from 2–3 per 100,000 person-years in 1980 to > 10 per 100,000 by 2000 [27]. Rising obesity is one key factor contributing to this rapid increase [28, 29]. Although obesity has also increased in Japan, the scale is more modest [9, 30, 31]. In addition, the increasing level of gastric acid secretion in the general Japanese population in the post-*H. pylori* era, driven by the natural decline in *H. pylori* infection [32] and the widespread use of eradication therapy [33, 34], may also contribute to EAC development. Consequently, Japan’s subsequent trend of increasing EAC, which began around 2010, is drawing attention. Our analysis using the most recent registry data reveals that both the number and incidence of EAC in Akita doubled across the 2010–2014 and 2020–2024 periods, indicating that the upward trajectory that began around 2010 has persisted. Still, the ASR among Japanese men in 2024 remained relatively low (2.1 per 100,000 person-years) compared with Western populations, suggesting that Japan has not yet entered the rapid growth phase seen in the West [27, 35, 36]. Continued surveillance of national EAC trends over the next 10–20 years is warranted.

In this study, the total number of EAC cases increased twofold between 2010–2014 and 2020–2024; however, early-stage EAC increased by 3.1-fold, exceeding the 1.5-fold increase in advanced-stage disease. This pattern likely reflects heightened recognition of EAC among endoscopists since around 2010 in this region [12], improving detection of early lesions. Efforts to further enhance early diagnosis remain important, particularly in improving overall outcomes. Importantly, the continued increase in advanced-stage EAC indicates that the rise is not merely due to earlier detection but reflects a true increase in disease burden.

A notable sex disparity was observed, consistent with established epidemiology [1, 2]. The male-to-female ratio was pronounced, and ASR increased significantly among men, whereas the small number of cases in women limited statistical evaluation. A striking sex difference was also observed in age at diagnosis: men developed EAC 12 years earlier than women, and most female cases were diagnosed at age ≥ 80. This finding mirrors previous European reports [37] and aligns with our recent study showing that Barrett’s esophagus, the precursor of EAC, develops 20–30 years earlier in men than in women [38]. This disparity may be partially explained by hormonal factors, such as estrogen, which may attenuate reflux-driven carcinogenesis and delay EAC development in women [39, 40]. Consistent with the period-based trend test and EAPC results, a supplementary joinpoint analysis did not identify a change in slope for the overall or male series, suggesting a sustained monotonic increase since 2010. Sex-specific visualization provided in Supplementary Fig. 3 underscores the pronounced male predominance and the limited precision of female estimates due to small numbers, reinforcing the need for larger, nationwide assessments and sex-informed screening strategies.

Our recent work has consistently shown that the risk of EAC increases markedly with Barrett’s esophagus length in the Japanese population [41–44]. Taken together, the present findings highlight the importance of integrating sex-specific risk patterns into future screening strategies. In particular, sex-specific thresholds for initial endoscopic evaluation and surveillance intervals may be warranted. These pronounced sex-related disparities in incidence and age at diagnosis have potential implications for cancer control policy in Japan and require confirmation using nationwide registry data.

Although this study provides up-to-date, population-based insights from a single prefecture, the findings likely reflect broader national trends. Prior reports suggest that the timing of EAC increases in Akita corresponds with trends observed in other regions of Japan (approximately 2005–2010) [13–15]. For example, Saito et al. reported steadily increasing EAC incidence among men after 2005 using population-based registry data from Yamagata, Fukui, and Nagasaki [15]. Furthermore, a roughly similar trend was observed in other studies [13, 14]. Therefore, the trends documented in Akita through 2024 are expected to be representative of national patterns.

Other than the retrospective style and single-prefecture setting, the lack of clinical information on EAC patients is a potential limitation of this study, although this is an inherent feature of studies using registration databases. Specifically, the mechanisms underlying the observed increase in EAC remain unclear due to the lack of information on potential risk factors, such as obesity, gastroesophageal reflux disease, or Barrett’s esophagus [45].

In conclusion, using the most recent high-quality registry data from Akita Prefecture, this study demonstrates that both the number and incidence of EAC doubled between 2010–2014 and 2020–2024. These findings confirm that the upward trend that began around 2010 has persisted. Continued monitoring of EAC incidence in Japan over the next several decades is essential.

## Supplementary Information

Below is the link to the electronic supplementary material.

Supplementary file1 (PDF 157 KB)

Supplementary file2 (DOCX 16 KB)

## Abbreviations

ASR: Age-standardized incidence rate
CI: Confidence interval
EAC: Esophageal adenocarcinoma
EAPC: Estimated annual percent change
EC: Esophageal cancer
ESCC: Esophageal squamous cell carcinoma
IR: Incidence rate
