# Supplementary material for: A steadily increasing trend in the incidence of esophageal adenocarcinoma in Akita Prefecture, Japan, through 2024
Source: J Gastroenterol. 2026 Apr 9;61(7):925–33. doi: 10.1007/s00535-026-02407-3 (PMC13283133; doi:10.1007/s00535-026-02407-3)
Supplement: Supplementary file 1 — Supplementary file1 (PDF 157 KB) [file 535_2026_2407_MOESM1_ESM.pdf]

Supplementary Figure 1

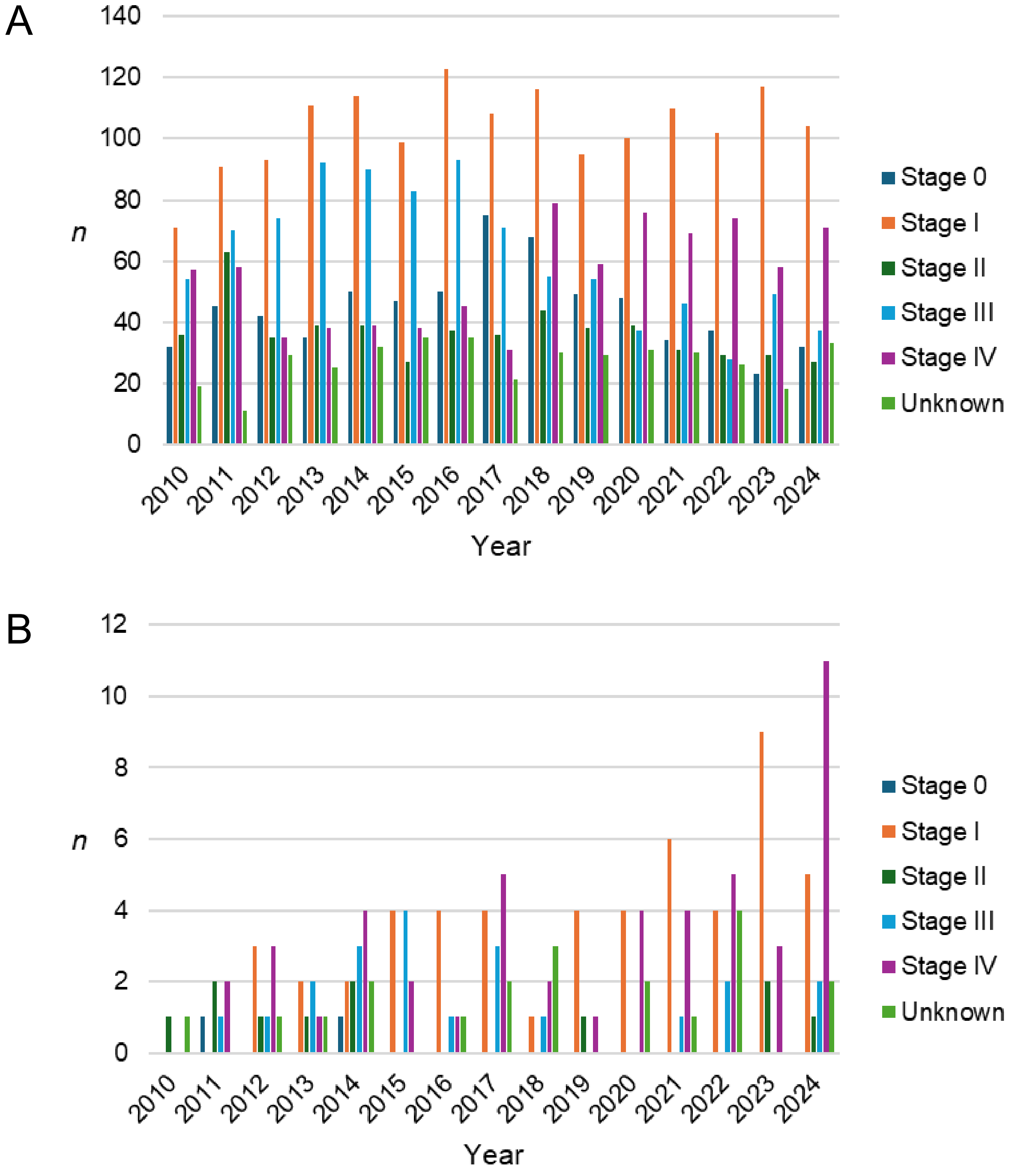

Supplementary Figure 2

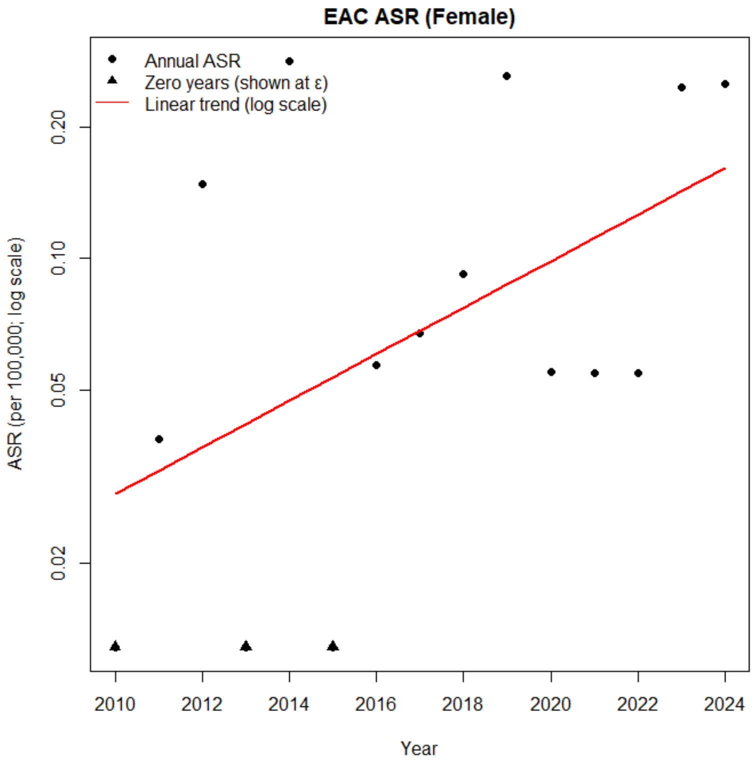

	EAPC, %/year	95% CI	*p*-value
**2010–2024**			
Female	12.01	0.33–25.05	0.0435

Supplementary Figure 3

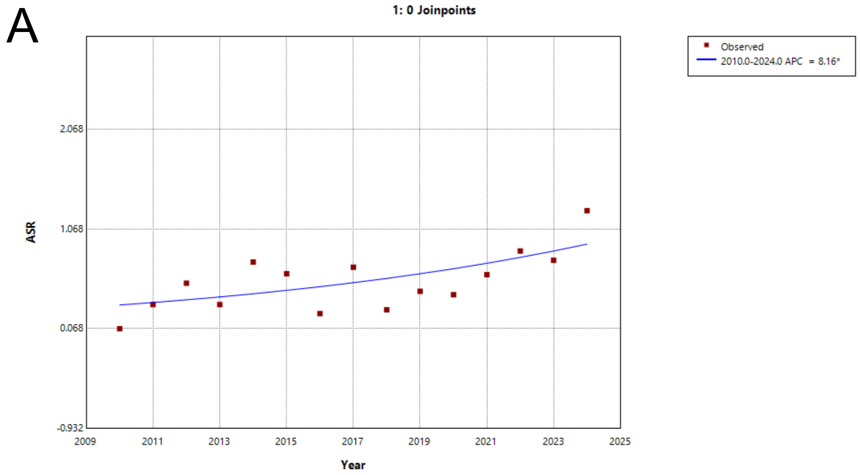

* Indicates that the Annual Percent Change (APC) is significantly different from zero at the alpha = 0.05 level.
Final Selected Model: 0 Joinpoints.

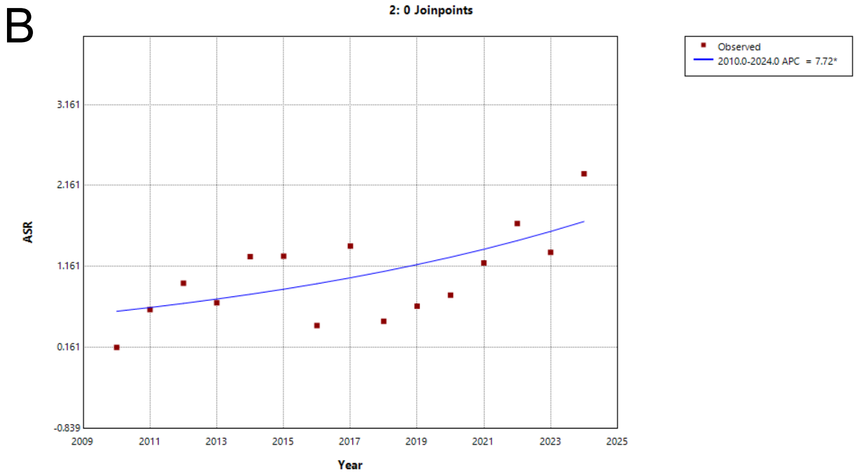

* Indicates that the Annual Percent Change (APC) is significantly different from zero at the alpha = 0.05 level.
Final Selected Model: 0 Joinpoints.

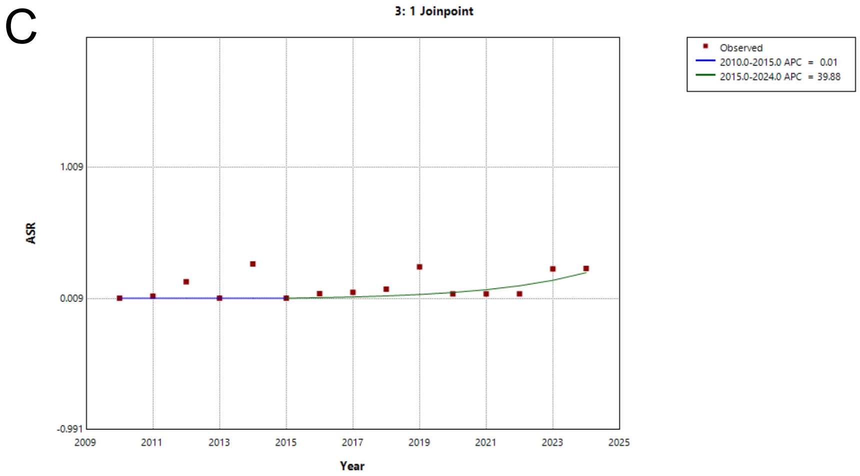

* Indicates that the Annual Percent Change (APC) is significantly different from zero at the alpha = 0.05 level.
Final Selected Model: 1 Joinpoint.
