# Supplementary material for: A steadily increasing trend in the incidence of esophageal adenocarcinoma in Akita Prefecture, Japan, through 2024
Source: J Gastroenterol. 2026 Apr 9;61(7):925–33. doi: 10.1007/s00535-026-02407-3 (PMC13283133; doi:10.1007/s00535-026-02407-3)
Supplement: Supplementary file 2 — Supplementary file2 (DOCX 16 KB) [file 535_2026_2407_MOESM2_ESM.docx]

**Supplementary Table 1. Cochran–Armitage trend test for the proportion of esophageal adenocarcinoma across three periods (2010–2014, 2015–2019, 2020–2024) in the total cohort and by sex.**

|  | χ² | *p*-value |
| --- | --- | --- |
| Total | 16.16 | <0.001 |
| Male | 12.07 | <0.001 |
| Female | 4.49 | 0.034 |
